# Nature Studies—V

**Published:** 1910-03-12

**Authors:** 


### The Practitioner's Relaxations.

### NATURE STUDIES—V.

By A MAJOR R.A.M.C.

St. Valentine's Day—Pairing Partridges—Field Fares, Hares and Rooks.

St. Valentine's Day is commonly regarded as bird-pairing time, and the beginning of spring; but when the days perceptibly begin to lengthen, after the coming of the New Year, the countrysides by many signs already show that winter is ending and springtime is drawing near.

The first six weeks of 1910 have been exceptionally interesting to me, for I have not seen the approach of English spring for several years. The year opened springlike on the downs. But for a few freezing days and a thin scatter of snow it has remained mild. The open weather and the consequent ease of food supplies rejoiced the birds. It seems to me that it has made them think untimely of mating. From the beginning there has been much singing. Thrushes, blackbirds, and robins have been in full chorus every morning outside my house; the starlings have sat and whistled and worked their wings excitedly on the tops of the lime-trees by the stable after their morning bath. A gentleman told me that he heard a linnet singing on January 8 in some gorse in a hollow of the downs, but I did not hear the lark's song till the very end of January. I wonder at this, for larks are usually singing as early as any bird.

I noticed symptoms of pairing amongst the partridges as early as January 20. On that date they were still in coveys, but the cock birds were chasing the hens, and by the end of the month the coveys had broken up into pairs. A covey is a family, so at first sight this pairing between the members of the covey looks as if there was constant inbreeding, a thing detrimental to any race. But Nature is wise in all her ways, and guards against inbreeding. The first pairing is but a preliminary. After the first set of pairs are formed there is rivalry and fighting among the cock birds. This results in re-distribution that is not quite complete till nesting begins. The main of the pairs are at their first essay in matrimony. It is possible that an old pair, if both birds have survived the shooting season, may remain together for another brood, but I have no evidence on this point. It would be interesting to find out to what extent birds remain paired for life. I feel sure that some birds, rooks and jackdaws for instance, to a great extent remain paired all through the year, but I think it probable that there is re-distribution of wives just before nesting begins. If it be true that there is a surplus of cocks amongst most species of birds, rivalry will lead to re-distribution. The question of surplus is of interest. There are statements to support it. Gamekeepers say that if the cock hawk of a nesting pair is killed the hen always finds a mate willing to assist in the work of building and even of incubation.

Some game preservers make a point of killing as many cock partridges as possible during the first week or two after pairing has commenced. They say that every widowed hen finds a fresh mate at once, and because there are fewer cocks there is less fighting, so earlier nesting. This points to surplus cocks.

Another reason for this killing of cocks is a belief that the old cocks are the first to pair. An old cock will not tolerate possible rivals near his chosen ground. He requires a wider space clear of other pairs. The young cock is less jealous. It follows that if there be many old cocks on a heavily-stocked estate there will be fewer pairs, and consequently fewer coveys in September than if the pairs have young cocks. By shooting the cocks of the first pairs old cocks are killed off and nests are more numerous.

The hares began to run early this year. On the downs and in fields adjoining there are vast numbers. Since the first fortnight of the year I have seen them running daily. It is a curious sight. The doe lopes along the face of the down or across fallows and young wheat, three or four jacks in single file follow close. She goes on and on, turning now one way now another, describing large irregular circles. At times the whole party stops and feeds till some amorous jack tries to press attentions, then off goes the doe again and all her followers after her. Now and again when the party stops two of the jacks will sit up fronting one another and appear to box at one another, or there may be battle royal in which rivals jump over one another and cut flecks of hair out of one another with blows of the hind legs. Sometimes upwards of a dozen hares may be in one party.

There was a superstition amongst the country people, perhaps there is still, that a doe hare in young cannot be caught by a greyhound. I have heard men speak of them as "witch hares." The origin of this belief is that the constant exercise they take at this season trains the hares. Both jacks and does get into hard condition and run before greyhounds with a speed and staunchness that they never show in the autumn.

The fieldfares are still here, but are on their way to the north I suppose. I find flocks now and again, but they do not stay in one place. The peewits were in flocks on Valentiue's Day, and gave no sign of pairing. That seems to me late, for peewits have eggs by the middle of March. However, on February 19 I saw two pairs on nesting ground chasing rooks and flying "peewiting" round intruders as is the manner of them in their nesting time. On February 22 after dark I heard a flock calling in the fields before my house, but in a few days the flocks will have resolved into scattered pairs.

The rooks were examining their old nests with a view to repairs at the end of January, but I have not yet seen any carrying in sticks.

On February 13 I saw jackdaws, paired, chattering round the holes in some gnarled old trees in which they breed.

